# Experimental study of the anti-tumour activity and pharmacokinetics of arctigenin and its valine ester derivative

**DOI:** 10.1038/s41598-018-21722-1

**Published:** 2018-02-19

**Authors:** Enbo Cai, Xingzhuo Song, Mei Han, Limin Yang, Yan Zhao, Wei Li, Jiahong Han, Shumei Tu

**Affiliations:** College of Chinese Medicinal Material, Jilin Agricultural University, Xincheng Street No. 2888, Changchun Shi, Jilin province, 130118 China

## Abstract

Arctigenin (ARG) is a functional active component that has important physiological and pharmacological activities. The anti-tumour and anti-inflammatory activities of ARG show good potential for application and development, but this material has the defect of low water solubility. In this experiment, the valine derivative of ARG (ARG-V) was designed and synthesized to overcome this disadvantage. The ARG amino acid, EDCI and DMAP were raw materials in the addition reaction, with a molar ratio of 1:2:2:0.5. The yield of ARG-V was up to 80%. ARG-V has strong anti-tumour activity *in vivo* and *in vitro*. The inhibitory rate of ARG-V was 69.2%, with less damage to the immune organs and different degrees of increased serum cytotoxicity. Moreover, the pharmacokinetics of ARG following oral administration and ARG-V following oral administration in rats were also studied. The C_max_ and AUC values of ARG-V showed significant differences compared to ARG. The relative bioavailabilities of three doses of ARG-V compared to ARG were 664.7%, 741.5% and 812.9%. These pharmacokinetic results may be useful for further studies of the bioactive mechanism of ARG and provide a theoretical basic for clinical use.

## Introduction

Hepatocellular carcinoma (HCC) is the third most common cause of cancer-related death in the world, with many new cases appearing every year. In some areas of Asia, HCC is the most common cause of death^1^. Two main treatment options for HCC are hepatic resection and liver transplantation^2^. However, some studies have shown that many patients relapse within five years after surgical treatment^3^. As a result, chemotherapy has been selected as a major means of treatment. However, patients with an advanced stage of cancer are rarely cured, and meanwhile, chemotherapy has many side effects, including pain, anorexia, cachexia, impaired taste, alopecia, nausea, vomiting, dehydration, mucositis, depression and anxiety^4^. Therefore, in recent years, increasing numbers of natural medicines with good anti-cancer efficacy and low toxicity have been found.

Nitrite is widely used as a food additive, especially in meat production^5^. Consuming excessive amounts of nitrite results in a long list of harms to public health. When nitrite reacts with secondary amines and amides in the stomach, nitrosamine, a strong carcinogen, is synthesised^6^. Furthermore, nitrite interferes with the oxygen transport system in the body by converting haemoglobin to methemoglobin in blood^7^. Therefore, the removal of nitrite *in vivo* is helpful to a certain degree in the prevention of cancer.

Amino acids are organic compounds that form proteins and are related to the activities of life. Amino acid molecules have been introduced into anticancer drugs as an endogenous substance from the organism that can increase selectivity for tumour cells, increase the solubility of drugs and decrease the toxicity to normal cells. Amino acid derivatives have been widely used in anticancer drugs all over the world^8^.

The pharmacokinetics of traditional Chinese medicines is based on a dynamics principle, involving the study of the dynamic variation of the *in vivo* absorption, distribution, metabolism and excretion of the active ingredients and components of Chinese medicine. It is a marginal discipline that uses mathematical analysis to obtain quantitative descriptions^9^.

Arctigenin (ARG) is a bioactive constituent obtained from the dried seeds of a traditional Chinese medicine, *Arctium lappa* L., that is widely studied by domestic and foreign researchers. ARG has also been indicated to possess diverse pharmacological activities, including anti-cancer^10–15^, anti-oxidative^16,17^, anti-inflammatory^18–22^ and anti-HIV activities^23–26^. Unfortunately, ARG cannot be fully absorbed *in vivo* because of its poor solubility. Amino acid prodrugs are known to be useful for improving the aqueous solubility of sparingly water-soluble drugs^27^.

Previous studies in our laboratory have shown that ARG-V has better solubility and nitrite removal activity than ARG^28^. On this basis, their pharmacological activities and their impact on immune function were compared in H_22_ tumour-bearing mice. In addition, the pharmacokinetics of ARG and ARG-V were studied. The results provide a reference for the further research and development of ARG.

## Results

### Preparation of ARG-V

**Figure 1:**
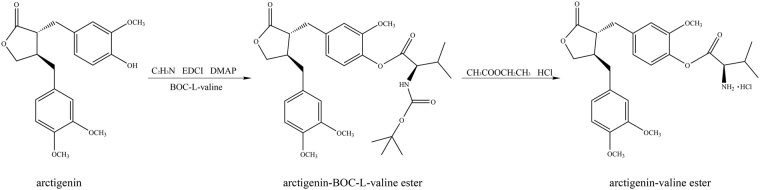
The synthesis of ARG-V.

Our previous study provided a method to synthesize the valine ester derivative of ARG, but here, we provide an improved method to synthesize this derivative after further optimization of the method. The optimum reaction time and the solvent were selected by experiment, and the yield of ARG-V was up to 80%. The synthesis of ARG-V is shown in Fig. 1.

### Synthesis of compound

4-(((3 R)-4-(3,4-dimethoxybenzyl)-2-oxotetrahydrofuran-3-yl))-2-methoxyphenyl.

2-amino-3-methylbutanoate hydrochloride (ARG-V).

White solid (80%), UV λ_max_(MeOH): 221 nm. HRESIMS m/z: 472.2340 [M-Cl]^+^ (Calcd for C_26_H_34_O_7_N:472.2335). ESI-MS m/z: 472.1 [M-Cl]^+^], ^1^H-NMR (300 MHz, CDCl_3_) δ: 6.70 (1d, 2.1 Hz, 1 H), 6.76 (d, 8.4 Hz, 1 H), 6.65 (dd, 8.4, 1.8 Hz, 1 H), 2.88–2.90 (m, 2 H), 2.60(m, CH), 6.49 (d, 2.1 Hz, 1 H), 6.95 (d, 8.1 Hz, 1 H), 6.54 (dd, 8.1, 1.2 Hz, 1 H), 2.50 (m, 2 H), 2.45 (m, 1 H), 3.86–4.05 (m, 2 H), 4.15 (m, 1 H), 2.64 (m, 1 H), 1.08 (d, 3 H), 3.82 (s, 3 H), 3.79 (s, 3 H), 3.69 (s, 3 H). ^13^C-NMR (300 MHz, CDCl_3_) δ: 137.51, 111.87, 150.60, 137.61, 121.53, 120.58, 34.57, 46.32, 178.46, 130.19, 113.21, 149.00, 147.85, 111.39, 122.36, 38.02, 41.02, 71.19, 167.24, 58.33, 29.88, 17.63, 17.28, 55.83, 55.80, 55.52.

### Solubility of ARG and ARG-V

According to Table 1, the water-solubility of the derivative was improved. This suggests that ARG-V was easier to dissolve in water than ARG.

**Table 1:** The solubility of ARG and ARG-V.

	Water	Acetonitrile	Cloroform	Acetonitrile:water 45:55	Ethyl acetate
ARG	almost insoluble	very soluble	freely soluble	very soluble	freely soluble
ARG-V	freely soluble	slight soluble	freely soluble	very soluble	almost insoluble

### *In vitro* anti-tumour experiment

The result showed that the IC_50_ values of ARG, ARG-V and V_C_ were 17.49, 2.19, and 0.69 mg/mL. The IC_50_ value of ARG-V was close to that of V_C_. Moreover, the clearance rate was also studied, as shown in Table 2. The result implied that the scavenging activity of ARG-V was better than that of ARG, and at a concentration of 4 mg/mL, the nitrite removal ability increased by more than 75% compared with ARG.

**Table 2:** Clearance rate of different volume of Vc, ARG and ARG-V on nitrite.

Clearance	Concentration (mg/mL)					
Sample	0. 125	0.25	0.5	1	2	4
V_C_	7.30 ± 0.06	17.23 ± 0.00	25.84 ± 0.03	54.49 ± 0.02	96.32 ± 0.00	97.75 ± 0.00
ARG	17.60 ± 0.02	23.41 ± 0.02	27.72 ± 0.03	31.09 ± 0.02	34.46 ± 0.02	39.14 ± 0.07
ARG-V	20.22 ± 0.00	27.90 ± 0.05	35.21 ± 0.07	45.51 ± 0.01	64.04 ± 0.01	69.10 ± 0.04

### ***In vivo*** anti-tumour experiment Effect of ARG-V on tumour growth, immune organ function, and the kidney index in mice bearing H_22_ hepatocellular carcinoma

As shown in Table 3, compared with the model group, the tumour weight was significantly reduced after treatment with ARG and with all doses of ARG-V (*P* < 0.01). However, compared with the middle dose group, the tumour weight of the ARG group was significantly increased (*P* < 0.01). It is obvious that ARG-V has a better inhibitory effect on tumour growth compared to ARG. Additionally, the tumour inhibition rate of the middle dose group was closest to that of the CTX group. There is no dependence effect between the doses. In addition, the spleen index and thymus index of mice in the CTX group were significantly lower than those in the model control group (*P* < 0.01). ARG and all doses of ARG-V increased the spleen index and the thymus index. However, compared with the ARG group, the middle dose ARG-V group shows a more significant difference (*P* < 0.01). Therefore, it can be concluded that ARG-V can inhibit the growth of tumours in tumour bearing rice, especially in the middle dose group. In addition, its side effects on the immune organs are lower than those induced by CTX.

**Table 3:** Effect of ARG-V on tumor growth and the immune organ, function, kidney index in H_22_ tumor-bearing mice.

Groups	Dosage (mg/kg)	Increase of body weight (g)	Tumor weight (g)	Inhibitory rate (%)	Thymus index (mg/g)	Spleen index (mg/g)
Normal	—	5.44	—	—	1.43 ± 0.19	3.63 ± 0.30
Model	—	7.11	1.79 ± 0.35	—	1.94 ± 0.16	6.89 ± 0.54
Positive	25	4.37	0.50 ± 0.11^**^	72.06	1.24 ± 0.20^**^	4.40 ± 0.40^**^
ARG	40	6.30	1.32 ± 0.34^**,##^	26.26	1.54 ± 0.26^**,#^	5.71 ± 0.50 ^**,##^
ARG-V	20	5.59	1.03 ± 0.19^**,##,a^	42.46	1.75 ± 0.24^*,##^	4.80 ± 0.42^**,#,aa^
ARG-V	40	4.94	0.55 ± 0.15^**,aa^	69.27	1.87 ± 0.21^##,aa^	4.53 ± 0.57 ^**,aa^
ARG-V	80	5.11	0.66 ± 0.17^**,#,aa^	63.13	1.77 ± 0.18^*,##,a^	5.34 ± 0.50^**,##^

Values are expressed as mean ± SD (n = 8).

**P < 0.01 as compared with the model group.^#^P < 0.05 as compared with the Positive group.

^##^P < 0.01 as compared with the Positive group.^a^P < 0.05 as compared with the ARG group.

^aa^P < 0.01 as compared with the ARG group.

### Effect of ARG-V on ALT, AST, BUN and CRE

Liver diseases are measured clinically via aspartate aminotransferase (AST) and alanine aminotransferase (ALT). These enzymes are widely distributed in both the liver and in other tissues. The kidney function change can be judged using BUN and CRE.

**Figure 2:**
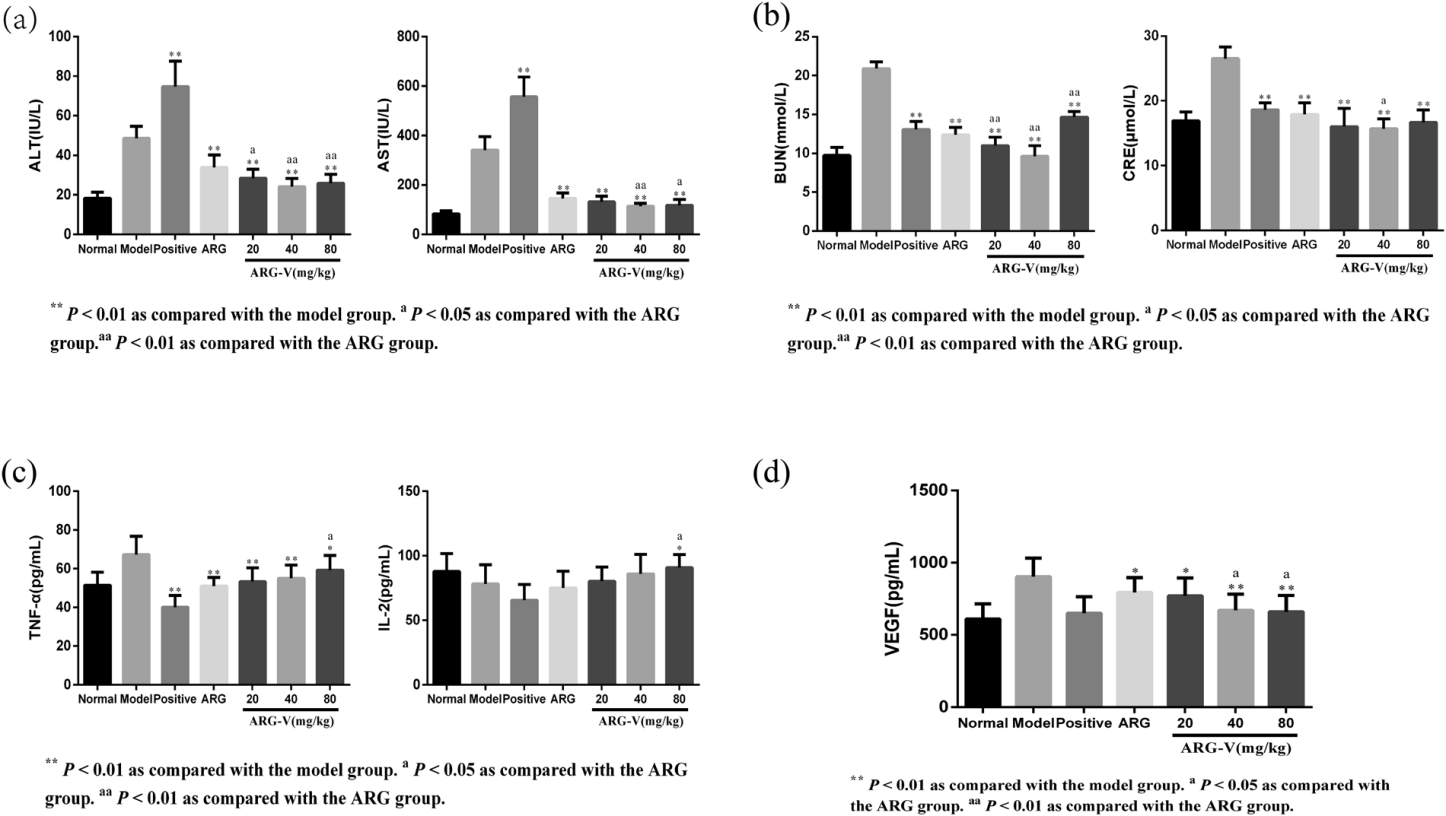
Effect of ARG-V on ALT, AST, BUN, CRE and serum cytokines.

According to Fig. 2(a), serum ALT and AST obviously increased in the CTX group compared to the model group (*P* < 0.01), while these indexes decreased after treatment with ARG and ARG-V, particularly in the middle and high dose ARG-V groups. In addition, the effect for the middle dose group was more significant than that for the ARG group (*P* < 0.01).

For the BUN and CRE indexes, shown in Fig. 2(b), the values in the ARG-V-treated groups decreased significantly compared to the model group (*P* < 0.01) and showed a significant difference compared to the CTX group. Among the groups, the middle ARG-V group was the closest to the normal group. However, there was no significant difference between the ARG group and the CTX group. These results suggest that ARG-V results in less damage to the liver and kidneys.

### Effect of ARG-V on serum cytokines

Serum cytokines also play an important role in antitumour activities. As shown in Fig. 2(c), the level of TNF-α in the CTX group significantly decreased compared with the model group (*P* < 0.01). Additionally, the concentration of serum TNF-α of the H_22_-bearing mice increased significantly after treatment with the three doses ARG-V compared with the CTX group (*P* < 0.01). The expression levels of IL-2 were significantly higher in the ARG-V groups compared to the tumour model group, particularly in the middle and high dose groups (*P* < 0.01) while it obviously decreased in the CTX group. The data shown in Fig. 2(d), clearly show that after treatment with CTX, serum VEGF decreased significantly, and all doses of ARG-V also decreased the serum VEGF compared with the model group. Among the groups, the middle and high dose groups showed no significant difference compared to the CTX group.

### Method validation of Pharmacokinetics

The regression equation was Y = 1. 7926X + 0.0006 (R^2^ = 0.9999), with the peak area ratios of ARG to the IS on the vertical axis and the blood concentrations on the horizontal axis.

The results indicated that the inter-day and intra-day precision of the three concentrations of ARG are between ~3.57 and 9.71% and the inter-day and intra-day accuracy are between ~3.17 and 5.36%.

The recovery of ARG was ~72.94–74.81%. The RSD was less than 15%. The method has good repeatability, although it does not have a high recovery. The recovery of the IS was 76.14%, and the RSD was 5.88%.

The stability of the three concentrations of ARG placed at room temperature for 12 hours, in a refrigerator (−20 °C) for a month, and after three repeated freeze-thaw cycles showed good results. The actual value of ARG is within 0.15 of the prepared value.

### Pharmacokinetic studies

**Figure 3:**
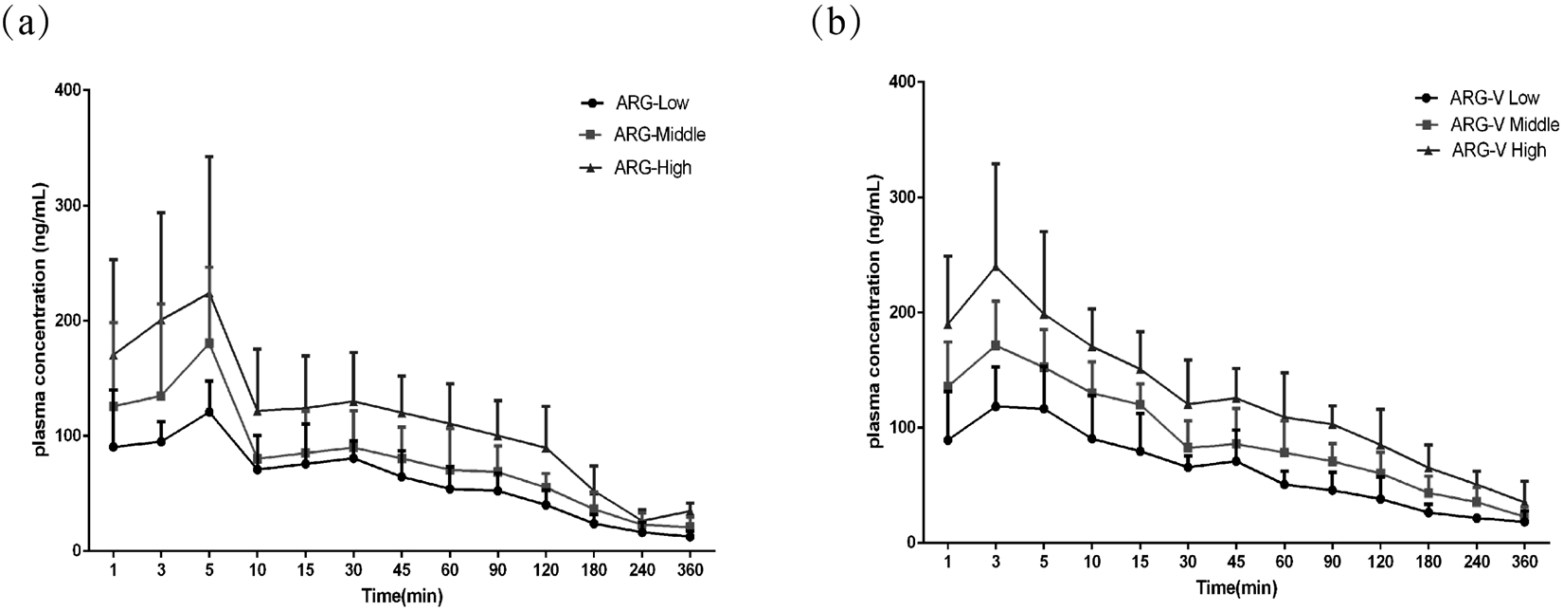
The concentration-time curve of ARG and ARG-V after oral administration to rats.

The pharmacokinetic profiles of ARG and ARG-V after oral administration to rats are illustrated Fig. 3, and the pharmacokinetic parameters are listed in Table 4. The measured blood concentration data were fitted using the “3P97” pharmacokinetic program. It can be clearly observed that the peak plasma concentration of ARG is attained within 5 minutes, while the peak plasma concentration of ARG-V is attained within 3 minutes. The AUC value of the three concentrations of ARG were 13687.547, 18971.963 and 25419.859 ng/mL, and the AUC value of three concentrations of ARG-V were 15162.740, 23446.734 and 34442.562 ng/mL. The relative bioavailability was 664.7%, 741.5%, and 812.9%.

**Table 4:** Pharmacokinetic parameters of ARG and ARG-V after intragastric administration to rats (X ± SD, n = 6).

Parameters	ARG			ARG-V		
	Low dosage	Middle dosage	High dosage	Low dosage	Middle dosage	High dosage
Ke(1/min)	0.006 ± 0.002	0.005 ± 0.001	0.006 ± 0.003	0.006 ± 0.002	0.005 ± 0.001	0.005 ± 0.001
Ka(1/min)	38.443 ± 16.895	6.550 ± 3.101	997.901 ± 344.563	39.298 ± 14.287	1479.817 ± 451.985	2520.489 ± 539.794
Lag time(min)	0.130 ± 0.037	0.020 ± 0.041	0.782 ± 0.187	0.305 ± 0.169	0.566 ± 0.175	0.102 ± 0.046
T_1/2(_Ka_)_(min)	0.018 ± 0.006	0.106 ± 0.064	0.0007 ± 0.0003	0.018 ± 0.007	0.0005 ± 0.0002	0.0003 ± 0.0001
T_1/2(_Ke_)_(min)	109.419 ± 45.656	128.821 ± 28.047	111.932 ± 26.243	124.462 ± 30.228	137.550 ± 31.454	147.547 ± 39.723
T_peak_(min)	0.227 ± 0.062	1.079 ± 0.564	0.012 ± 0.007	0.226 ± 0.089	0.009 ± 0.002	0.005 ± 0.003
C_max_(ng/mL)	86.584 ± 28.081	102.082 ± 25.953	157.403 ± 44.977	84.337 ± 16.993	118.148 ± 22.698	161.804 ± 19.393
AUC(ng/mL)*min	13687.547 ± 3050.502	18971.963 ± 5515.267	25419.859 ± 5497.001	15162.740 ± 2447.532	23446.734 ± 5967.979	34442.562 ± 4735.840
CL/F(s)mg/kg/min/(ng/mL)	0.004 ± 0.001	0.006 ± 0.003	0.009 ± 0.003	0.0008 ± 0.0001	0.001 ± 0.0003	0.001 ± 0.0002
V/F(c)mg/kg/(ng/mL)	0.646 ± 0.163	1.097 ± 0.118	1.423 ± 0.173	0.150 ± 0.031	0.215 ± 0.037	0.313 ± 0.028

## Discussion

Although ARG has many pharmacological activities, clinical studies have shown that it has low bioavailability. Therefore, it is necessary to modify ARG to form derivatives with better solubility and higher bioavailability by either chemical or biological methods^29^. Amino acid prodrugs are very useful for improving the aqueous solubility of sparingly water-soluble drugs^30^. Using the phenolic hydroxyl group and amino acid carboxyl group of ARG under proper conditions can induce the esterification reaction. In addition, previous studies have shown that the synthesis of the valine ester can improve the solubility and bioavailability of ARG^31^.

The esterification reaction is reversible, so control of the reaction time and temperature is extremely important. Esterification requires strict control of the reaction time to improve the yield efficiency and is favourable for industrial scale production. This study also found that after esterification for half an hour, the reaction reached thermodynamic equilibrium. The conversion of the reactant was above 98%, but its amino acid derivative product rate was low at that time. The yield was high when the reaction time was increased to an hour. Thus, it can be found that the degree of esterification depends not only on the conversion rate of ARG but also on the yield of ARG-V. The extent of the reaction increased with additional time and reached chemical equilibrium between 1 and 2 hours. The final determined optimal reaction time was an hour. At the same time, the esterification reaction is an exothermic reaction. With an increase in the reaction temperature, the conversion rate of ARG and its amino acid derivative production rate both decreased, so it is suitable for the esterification reaction to proceed in the conditions of an ice water bath. Meanwhile, the kind of solvent and the amount of solvent were also studied. DMSO, acetonitrile, dichloromethane, and tetrahydrofuran were tested as the reaction solvent. The results showed that with DMSO and tetrahydrofuran, the conversion rates of ARG and the yield were both low. When acetonitrile and dichloromethane were used as the reaction solvent, the conversion rates of ARG were 97.8%, but for dichloromethane, the yield of ARG-V was lower than that for acetonitrile. As a result, acetonitrile was selected as the reaction solvent. In addition, with increasing amount of solvent, the conversion rate of ARG gradually decreased due to the effect of the increased solvent volume on intramolecular collisions. The eventually determined amount of solvent for the reactants was a solvent ratio of 1:10 (g/mL). Thus, the chosen conditions were a reaction time of 1 h in an ice water bath, and the quantity of reactant (g) and the amount of solvent (mL) ratio was 1:10.

Studied have shown that BOC may influence the synthesis^32^. Thus, it is necessary to remove the BOC group, which is always removed under acidic conditions^33^. The trifluoroacetic acid method has primarily been adopted for this experiment. However, when alkali is used to neutralize the trifluoroacetic acid after removal of the BOC protecting group, the ester bonds of ARG and ARG-V are also easily ruptured. This method also difficult to operate and costly. Hydrochloric acid, a mineral acid, was later adopted, and the carbonyl oxygen of Boc was protonated by hydrogen, followed by dissociation and formation of the amino acid ester hydrochloride. This method may improve the efficiency and save operating costs.

Numerous methods for nitrite clearance have been reported, such as spectrophotometric^34^, chromatographic-mass spectrometric^35^, electrochemical^36^, and fluorescence methods^37^. Considering various factors, we decided to use diazo coupling spectrophotometry to perform this experiment^38^. The experimental result showed that the nitrite removal ability increased substantially because of the better solubility. On the other hand, it may be that ARG-V also has the ability to bind nitrite such that the nitrite content decreased, achieving the purpose of clearing nitrite. It has also been suggested that the structural transformation of arctigenin in clinical applications has important significance.

Recently, increasing numbers of Chinese natural medicines have been found to be anti-tumour agents^39^. These traditional medicines not only have remarkable anti-tumour effect but also have fewer side effects^40^. Our study has demonstrated that arctigenin has anti-tumour effects and that its amino acid derivative (ARG-V) exhibited dramatically improved pharmacological activity. ARG-V has a better inhibition effect on tumour growth in H_22_-bearing mice compared to arctigenin, without a dose-dependent effect. Moreover, ARG-V causes less damage to the immune organs, which many chemical drugs harm. Admittedly, serum cytokines also play an important role in anti-tumour activity. For example, many traditional medicines repress the angiogenesis level of VEGF in H_22_ hepatoma transplanted tumours, which plays a decisive part in anti-tumour activity^41^. Additionally, TNF-α is key to the defence against tumour cells through the induction of other immunoregulatory and inflammatory mediators. IL-2 also plays a vital role in anti-tumour activity and immune regulation. The results showed that after treatment with ARG-V, the serum TNF-α and IL-2 levels were significantly improved compared to the model group, while the serum VEGF obviously decreased, particularly in the middle dose group of ARG-V, which is the closest to the normal group. This experiment not only provides a new chemical process to improve the solubility of insoluble drugs but also lays the foundation for clinical applications and the development of arctigenin in the future.

A method for the quantitative analysis of the plasma levels of ARG in rats was established using a high performance chromatographic method (HPLC). This method has the advantages of high sensitivity, high specificity and high speed. It can be used for preclinical pharmacokinetic studies. First, the wavelength was determined. ARG dissolved in methyl alcohol or acetonitrile absorbs strongly at the wavelengths of 221 nm and 280 nm. However, endogenous substances in plasma also have strong absorption peaks at this wavelength, so finally, the latter was chosen. Different influencing factors, such as the proportion of the mobile phase and the chromatography column, were also studied. The accumulated evidence indicated that an ideal separation can be achieved when the mobile phase was an acetonitrile-0.1% solution of added formic acid (45:55). When combined with formic acid, a symmetric chromatograph peak was obtained for ARG and the IS. The Thermo BDSHYPERSIL C_18_ column (4.6 × 250 mm, 5 μm) showed the best resolution. Second, the method of bio-sample pretreatment was determined. Protein precipitation was accomplished by the addition of acetonitrile, and the use of plasma and acetonitrile at a mass ratio of 1:5 gives the best results. Meanwhile, centrifugation at a temperature of 4 °C precipitated the protein to the greatest degree. In addition, after redissolution with acetonitrile, the endogenous substances in the plasma did not interfere significantly with the determination of the samples compared to redissolution in the mobile phase. The supernatant was directly injected into the HPLC without filtration after high-speed centrifugation.

The administration method for a medicine is an important factor that influences the activity and effectiveness significantly. Oral administration is the most common method and is one of the oldest methods in history because it is simple, safe, economical and effective, and patients can take their own medicine by themselves. Some studies have shown that the solubility and oral bioavailability of arctigenin are both low, which is the dominant factor that restricts not only the development and utilisation of arctigenin but also the development and utilisation of Fructus Arctii as a medicinal and health care product. The pharmacokinetics of ARG by oral administration has been a hot area of research for several years^42^. Our research indicates that after Wistar rats were orally administered ARG and ARG-V, the AIC value indicated that the blood concentration-time curve could be fit with a one compartment model, and the weight was 1/c^2^. When three different dosages of ARG and ARG-V were delivered through oral administration, the area under the curve (AUC) did not show a linear relationship with the dosage of medication, indicating that the oral absorption process is a nonlinear dynamic process and that *in vivo* absorption may be saturated. In addition, the results showed that each concentration of ARG-V exhibited a fast absorption phase and a lasting elimination phase compared to ARG. ARG-V can improve the disadvantages of ARG, such as the fast elimination or frequent administration needed to maintain an effective concentration of ARG. Our experiment compared the pharmacokinetics of ARG and its valine ester derivative and proved that the arctigenin amino acid ester derivative has been converted into the original medicine and continues to function.

Furthermore, Liu^43^ carried out a toxicity study of ARG in Beagle dogs. There was no overt toxicity in the 60 mg/kg group except the stimulus response at the administration location, but repeated subcutaneous injection of large doses of ARG can induce injury to the liver and biliary duct and strengthen the reaction to the stimulus caused by PEG in dogs. Masafumi Ikeda^44^ has studied the pharmacokinetics of ARG in patients with advanced pancreatic cancer refractory to gemcitabine. The results showed that ARG in patients exists as arctigenin glucuronide (AGG), and second peaks of ARG and AGG were observed, indicating enterohepatic circulation. These survey results laid a foundation for further study on the pharmacokinetics of different administration routes and the speciation of ARG-V.

By the comparison and analysis of these parameters, ARG-V was absorbed rapidly and eliminated slowly compared to ARG, and the bioavailability of ARG-V was significantly higher than that of ARG. These results are similar to those from the *in vivo* anti-tumour experiment, in which the inhibition rate of ARG-V was much better than that of ARG. It is speculated that ARG-V is water soluble and easy to absorb via active transport by the amino acid connection.

## Materials and Methods

### Chemicals and reagents

The arctigenin standard was purchased from Chengdu Pure Chem-Standard Co., Ltd. ARG and ARG-V (HPLC purity >98%) were synthesized based on a previous study. Isobergapten (purity >98%), used as an internal standard (IS), was obtained from Nanjing Spring & Autumn Biological Engineering Co., Ltd. Cyclophosphamide (CTX) was obtained from Shanghai Hualian Pharmaceutical Co., Ltd. Glutamic oxalacetic transaminase (AST), glutamic-pyruvic transaminase (ALT), creatinine (Cr) and urea nitrogen (BUN) detection kits were purchased from the Nanjing Jiancheng Bioengineering Institute. Vascular endothelial growth factor (VEGF), tumour necrosis factor-α (TNF-α), and interleukin-2 (IL-2) detection kits were obtained from Shanghai Le Sheng Biotechnology Co., Ltd. HPLC-grade methanol and acetonitrile were purchased from Fisher (USA). HPLC-grade formic acid was purchased from Sigma-Aldrich (56302-10*1ml-F). Other chemicals were all of analytical grade.

### Animals

SPF (specific pathogen free) grade, male ICR mice with body weights ranging from 19 to 21 g were obtained from the Laboratory Animal Quality Testing Center of Jilin Province. The ICR mice were divided into 7 groups as follows: the normal group, the model group, the cyclophosphamide (CTX) group, the ARG group, the high dose ARG-V group, the middle dose ARG-V group and the low dose ARG-V group. H_22_ hepatocarcinoma cells were purchased from the Institute of Biochemistry and Cell Biology, CAS.

Male Wistar rats (8 weeks, 230–250 g) were purchased from Liaoning Changsheng Biotechnology Co., Ltd. (Certificate No. SCXK-2015-0001) and acclimated to the laboratory environment for 1 week.

The animal procedures were performed in accordance with the National Institutes of Health Guide for the Care and Use of Laboratory Animals and approved by the Animal Ethics Committee of the China Academy of Chinese Medical Sciences.

### Preparation of ARG-V

The following reaction conditions were used to produce arctigenin. An amino acid: EDCI: DMAP ratio of 1:2:2:0.5 was used, and the mixture was dissolved in acetonitrile for 1 h at 0 °C. Finally, the mixture was evaporated under reduced pressure to produce a yellow powder. The yellow powder was added to water, washed with stirring, dried, and then concentrated by lyophilisation to produce a crude white product. The crude products were separated by column chromatography with YMC reverse phase packing with an acetonitrile/water (55:45) mixed solvent elution. The required components were collected, the organic solvent was vacuum evaporated, and the resulting products were freeze-dried. The final product was a white pulverous compound.

Then, the removal of the BOC protecting group was carried out. First, the white powder compound was placed in a test tube with ethyl acetate in an ice water bath. Hydrogen chloride gas was added to the test tube and allowed to react for approximately 1 h. After the reaction was complete, the organic solvent was vacuum evaporated, and ARG-V was finally obtained.

### Solubility experiments

Solubility is a physical property of the drug. The reaction was performed according to the Chinese Pharmacopoeia, 2015 edition.

### *In vitro* anti-tumour experiment

Our team has studied the nitrate scavenging activity of ARG and ARG-V using diazo coupling spectrophotometric method^28^.

### ***In vivo*** anti-tumour experiment

To prepare the ascitic tumour-bearing model, 0.2 mL of the cell suspension (1 × 10^5^ cells/mouse) was injected into the shoulder of the right limb subcutaneously in each mouse. Twenty-four hours after the tumour inoculation with H_22_ cells, the ICR mice were divided into 7 groups as follows: the normal group, the model group, the cyclophosphamide (CTX) group, the ARG group, the high dose ARG-V group, the middle dose ARG-V group, and the low dose ARG-V group. The positive control group received CTX at a dosage of 25 mg/kg by intraperitoneal injection. The model group and the normal group were treated with the same amount of normal saline by P.O. once a day. The mice were given an ARG suspension solution mixed with sodium carboxymethyl cellulose at a daily dose of 40 mg/kg, and ARG-V dissolved in saline at different dosages was administered orally. The mice in each group were treated continuously for 14 days. Before and after each drug administration, the size of the tumours were measured, and the weights of the mice were recorded. Twenty-four hours after the last administration, the mice were killed by cervical dislocation, and blood samples were collected from the eyes of the mice. The tumour, spleen, and thymus of the mice were taken out and weighed on an electronic balance.

### Measurement of aspartate transaminase (ASL), alanine aminotransferase (ALT), creatinine (CRE) and blood urea nitrogen (BUN)

AST and ALT are clinical indexes of liver function, used in medicine to judge whether the liver is damaged. BUN and CRE are used to judge whether the kidney is damaged. They were analysed by a commercially available reagent kit according to the instructions.

### Measurement of cytokines

The serum levels of the cytokines IL-2, IL-6, VEGF and TNF-α were analysed using a commercially available ELISA kit according to the manufacturer’s instruction.

### Preparation of the calibration solution, IS solution and quality control (QC) samples

The standard stock solutions of ARG and IS were prepared in acetonitrile at a concentration of 10 mg/mL and 300 μg/mL, respectively. A suitable amount of the stock solution was taken and diluted with 10% acetonitrile by the serial dilution method to obtain working solutions (1000, 100, 10, 1 and 0.1 µg/mL) before use. The stock solution of the IS was diluted to 3 μg/mL.

The ARG stock solution was diluted with plasma to make quality control (QC) samples, and the QC samples were prepared at three concentrations of low, medium and high (0.02, 0.1, and 2 μg/mL).

### Plasma sample pretreatment

After the frozen plasma thawed, a 0.2 mL rat plasma sample was placed in an Eppendorf tube containing 5 µL of the IS working solution and 1 mL of acetonitrile with 0.1% formic acid and mixed evenly with a turbine mixer for 3 minutes. It was then separated by centrifugation (12000 rpm, 10 min, 4 °C). The supernatant was withdrawn and evaporated to dryness under N_2_. The dry residue was reconstituted by mixing it with 50 μL of acetonitrile for 1 minute and separated by centrifugation (16000 rpm, 5 min, 4 °C), and a 20 μL aliquot was injected into the HPLC system for analysis.

### Pharmacokinetics of ARG in normal rats after oral and intravenous administration

The rats received ARG and ARG-V by oral administration. Blood samples were obtained via the oculi chorioideae vein before oral administration and at 1, 3, 5, 10, 15, 30, 45, 60, 90, 120, 150, 180, 240, and 360 min after administration. The whole blood samples were separated by centrifugation (8000 rpm, 10 min), and the plasma was obtained and stored at −20 °C until analysis.

### Method validation

Analytes were separated on a Thermo BDS HYPERSIL C_18_ column (4.6 × 250 mm, 5 μm) at a flow rate of 1.0 mL/min. The column was maintained at 30 °C. The mobile phase consisted of acetonitrile and 0.1% formic acid water (45:55 v/v). Detection was done at a wavelength of 280 nm.

The stock solution of ARG was diluted with rat plasma to obtain the plasma samples (2.5, 1, 0.5, 0.25, 0.1, 0.05, 0.025 and 0.01 μg/mL). Calibration curves from 0.01 μg/mL to 2.5 μg/mL were generated by plotting the peak area ratios of ARG to the IS against the corresponding theoretical spiked concentration. Correlation coefficients and the regression equation were obtained.

The accuracy and precision were checked using QC samples with working solution concentrations of 0.02, 0.1 and 2 μg/mL, which were analysed on three consecutive days. The intra-day precision was determined by assaying standard solutions of the analyte at different times during the same day. The inter-day precision was determined by assaying standard solutions of the analyte over three consecutive days. The concentration of each sample was determined using a calibration curve.

Plasma samples without the ARG stock solution and the IS solution were pretreated; then, the supernatant was obtained to make samples with three different ARG concentrations (0.02, 0.1 and 2 μg/mL) using the ARG stock solution. After evaporating to dryness under N_2_ and reconstituting the dry residue, a 20 μL aliquot was injected into the HPLC system. The peak area (B1) was recorded. QC samples were prepared at low, medium and high concentrations and processed with the method described above, and the peak area was obtained (B2). The peak area ratios of the two treatments of each concentration were obtained to calculate the extraction recovery. The same method was used to calculate the extraction recovery of the IS solution.

The QC samples were kept at room temperature for 12 h, and freeze-thaw cycles were repeated 3 times. The QC samples were kept at −20 °C for 30 days.

### Data analysis

The values are presented as the mean ± standard deviation (SD). The concentration-time curve was drawn, and data were processed using the 3P97 software.
